# Crystal Structures of Human C4.4A Reveal the Unique Association of Ly6/uPAR/α-neurotoxin Domain

**DOI:** 10.7150/ijbs.39919

**Published:** 2020-01-30

**Authors:** Yunbin Jiang, Lin Lin, Shanli Chen, Longguang Jiang, Mette C. Kriegbaum, Henrik Gårdsvoll, Line V. Hansen, Jinyu Li, Michael Ploug, Cai Yuan, Mingdong Huang

**Affiliations:** 1 State Key Laboratory of Structure Chemistry, Fujian Institute of Research on the Structure of Matter, Chinese Academy of Sciences, Fuzhou, Fujian, 350002, China.; 2 University of Chinese Academy of Sciences, Beijing 100049, China.; 3 Institute of Oceanography, Minjiang University, Fuzhou, 350108, China.; 4 College of Chemistry, Fuzhou University, Fuzhou, Fujian, China.; 5 Finsen Laboratory, Rigshospitalet, DK-2200 Copenhagen N, Denmark.; 6 Biotech Research and Innovation Centre (BRIC), University of Copenhagen, DK-2220 Copenhagen N, Denmark.; 7 College of Biological Science and Engineering, Fuzhou University, Fuzhou, Fujian, 350116, China.

**Keywords:** C4.4A, uPAR, three-fingered fold, LU-domain.

## Abstract

Ly6/uPAR/α-neurotoxin domain (LU-domain) is characterized by the presence of 4-5 disulfide bonds and three flexible loops that extend from a core stacked by several conversed disulfide bonds (thus also named three-fingered protein domain). This highly structurally stable protein domain is typically a protein-binder at extracellular space. Most LU proteins contain only single LU-domain as represented by Ly6 proteins in immunology and α-neurotoxins in snake venom. For Ly6 proteins, many are expressed in specific cell lineages and in differentiation stages, and are used as markers. In this study, we report the crystal structures of the two LU-domains of human C4.4A alone and its complex with a Fab fragment of a monoclonal anti-C4.4A antibody. Interestingly, both structures showed that C4.4A forms a very compact globule with two LU-domain packed face to face. This is in contrast to the flexible nature of most LU-domain-containing proteins in mammals. The Fab combining site of C4.4A involves both LU-domains, and appears to be the binding site for AGR2, a reported ligand of C4.4A. This work reports the first structure that contain two LU-domains and provides insights on how LU-domains fold into a compact protein and interacts with ligands.

## Introduction

Proteins with Ly6/uPAR/α-neurotoxin domain (LU-domain), also named three-fingered protein domain or TFPD, are widespread in the animal kingdom and mainly comprises either secreted or glycosyl-phosphatidylinositol (GPI) anchored single domain proteins with diverse biological functions 1. The hallmark of a prototypical LU-domain is 8 conserved cysteine residues engaged in a defined disulfide-bonding, which forms a compact cysteine-rich knot (palm) projecting three extended loops (fingers) stabilized by 5-6 β-strands 2-4. This domain fold has been extensively utilized in the evolution of a variety of snake venom toxins targeting acetylcholine receptors (*e.g.,* α-neurotoxins), acetylcholine esterases (*e.g.,* fasiculin), L-type calcium channels (*e.g.,* calciseptins) or targeting cell membranes (*e.g.,* cardiotoxins) 4. In a coral snake, up to 95% of its venom toxin are TFP toxins 5. In mammals, secreted or GPI-anchored single LU-domain-containing proteins are also important mediators of diverse aspects of physiology including inhibiting autologous complement activation (CD59) 6, modulating neuronal acetylcholine receptors (Lynx1 and SLURP1) 4, 7, and securing efficient intravascular triglyceride hydrolysis by trafficking and stabilizing lipoprotein lipase (GPIHBP1) 8-10.

Notwithstanding the prevalence of single LU-domain-containing proteins in the animal kingdom, there are only a few examples where two or more LU-domains forming the functional unit. In venomous snakes, certain neurotoxins evolved unique functions *via* homodimeric assembly using either non-covalent interactions (*e.g.,* κ-bungarotoxin and haditoxin) 11, 12 or covalent disulfide linkage (*e.g.,* iriditoxin and α-cobratoxin) 13, 14. In mammals, CD59 forms dimer, and further to oligomers, in lipid rafts of cell surface and induce intracellular Ca^2+^ response 15. Significantly, a small gene cluster located on chromosome 19q13 in humans encodes GPI-anchored proteins containing 2-4 consecutive LU-domains (*e.g.,* uPAR, C4.4A, Haldisin, TEX101, CD177, and PINLYP) 2, 16. These multiple LU-domain-containing membrane proteins evolved diverse important roles. For instance, uPAR plays important roles in focalizing plasminogen activation on cell surfaces and regulating cell motility and immune response 16. The elevated soluble uPAR level in plasma is associated with incident acute 17 or chronic kidney disease 18, cardiovascular disease 19, and human cancer 20. The CD177 mediates neutrophil endothelial transmigration 21, 22, and its overexpression is associated with chronic myeloproliferative disorders 23. TEX101 regulates fertility 24. C4.4A and Haldisin define stages of squamous epithelial differentiation 25-27.

Despite the clear functional importance of these multiple LU-domains proteins, their three-dimensional structures remain largely unexplored with a single exception. The urokinase-type plasminogen activator receptor (uPAR) is a GPI-anchored membrane protein containing three LU-domains (DI, DII and DIII) and several crystal structures have been solved for this founder of the LU-domain protein family 28-32. The intermolecular assembly of all three LU-domains in uPAR *via* β-sheet interactions creates a large central hydrophobic ligand-binding cavity that mediates the high-affinity binding of its primary ligand, the serine protease urokinase-type plasminogen activator. Biophysical studies have shown that this interdomain assembly in uPAR is highly flexible and that this has biological relevance 33, 34. Restricting this internal flexibility by introducing an interdomain disulfide bond between the DI and DIII traps uPAR in a closed conformation, which increases its affinity for its second ligand, Vitronectin 33, 35. From a translational perspective, this domain flexibility also proved essential for the development of a small 9-mer peptide targeting an intermediate conformation in uPAR 28, 36 and this assisted its further maturation into a PET-probe currently used for non-invasive imaging of uPAR expression in patients with malignant solid tumors 37-39. Moreover, the dimer of uPAR isoform 2 was reported to induce kidney diseases in mice 40.

Prompted by the close relationship between LU-domain flexibility and function of uPAR, we decided to solve the crystal structure of another protein containing multiple LU-domains to gain further insight into the structural versatility of this fold. We chose to focus on C4.4A (encoded by *LYPD3*), which contains two LU-domains followed by a mucin-type region rich in serine, threonine and proline (STP-rich region) and a C-terminal GPI-anchor 41, 42. No well-defined function has yet been assigned to C4.4A, but circumstantial evidence suggests that it could play a role in cell adhesion, migration and invasion through established interaction with laminins 43, integrins and MMP14 44, 45, and/or Anterior Gradient 2 (AGR2) 46. Nonetheless, expression of C4.4A is strictly regulated under normal homeostatic conditions as it represents a robust biomarker for the presence of *stratum spinosum* in stratified squamous epithelia of the skin and for squamous differentiation of epithelia in other organs such as esophagus, vagina, oral cavity, and rectum 27, 42, 47. Along the same lines, squamous metaplasia of bronchial epithelia (not yet a malignant lesion) is strictly correlated with the emergence of C4.4A expression 48. Consequently, high expression levels of C4.4A predicts poor prognosis for patients with pulmonary adenocarcinoma but not for those with squamous cell carcinoma 20, 49, 50. Similar findings have been reported in other solid cancers in *e.g.*, breast 51, bladder 52, 53, colon 54, 55, and esophagus 56, 57. Based on these findings, there is a strong interest in studying C4.4A in various pathological conditions and new experimental tools are being developed to accomplish this—such as C4.4A-deficient mouse models 53 and antibody drug conjugates targeting C4.4A 58. With this study, we seek to gain structural insights into how the LU-domains in C4.4A are organized and how C4.4A recognizes ligand.

## Results

### Challenges in structural determination of C4.4A

Recombinant human C4.4A was expressed in *Drosophila* S2 cells. This recombinant protein contains, at its C-termini, a purification tag (uPAR DIII) to facilitate the capture and purification of the protein 59. The STP-rich region of C4.4A is heavily glycosylated containing 15 putative O-linked glycosylation sites 42, posing major difficulty for the crystallization of intact C4.4A. We thus removed the STP-rich region and the purification tag by limited proteolysis with chymotrypsin to obtain the N-terminal region containing the two LU-domains (DI and DII) of C4.4A (residues 1-201), which was then purified to high homogeneity, and grown into well diffracting crystals of C4.4A (2.4 Å) at pH 3.6 60. Structural determination from these C4.4A crystals using molecular replacement (MR) proved difficult due to the low sequence conservation amongst published structures of single LU-domain (*e.g.*, the two LU-domains in human C4.4A share only 30% and 28% sequence identity with the DII of uPAR). Single-wavelength anomalous dispersion phasing using a biosynthetically selenomethionyl labeled C4.4A (yielding ~70% Se-incorporation in Met) was unsuccessful due to poorly diffracting SeMET crystals (>4 Å). Traditional multiple isomorphous replacements (MIR) or phasing with sodium bromide 61 were also tried, but in all cases, the crystals either lost their diffraction upon soaking or did not give clear solutions of heavy atom positions.

The complex between C4.4A and the Fab fragment of 11H10 was purified by size exclusion chromatography and yielded well-diffracting crystals of C4.4A:Fab (11H10). The Fab fragment was positioned into the crystal by MR, and the Fo-Fc map now revealed electron density for C4.4A. Extensive manual building, together with iterative refinement, finally yielded crystal structures of both the C4.4A:Fab complex and C4.4A with good statistics. The structure of C4.4A was refined to 2.59 Å with R-factor and R-free of 0.2063 and 0.2503, respectively; 92.8% residues in favored Ramachandran region (Table 1). Most residues of the C4.4A structure are well supported by electron density maps, except for residues 95-99 and residues 92-101—a glycosylated linker region between the DI and DII—which were consequently not modelled in the structure. The structure of C4.4A:Fab complex was refined to 2.75 Å with R-factor and R-free of 0.1963 and 0.2555, respectively; 95.3% residues in favored Ramachandran region (Table 1). Residues 89-103 of C4.4A molecule between DI and DII are also not modelled.

### Crystal structure shows that the two LU-domains in C4.4A forms a compact and globular unit

**Figure 1:**
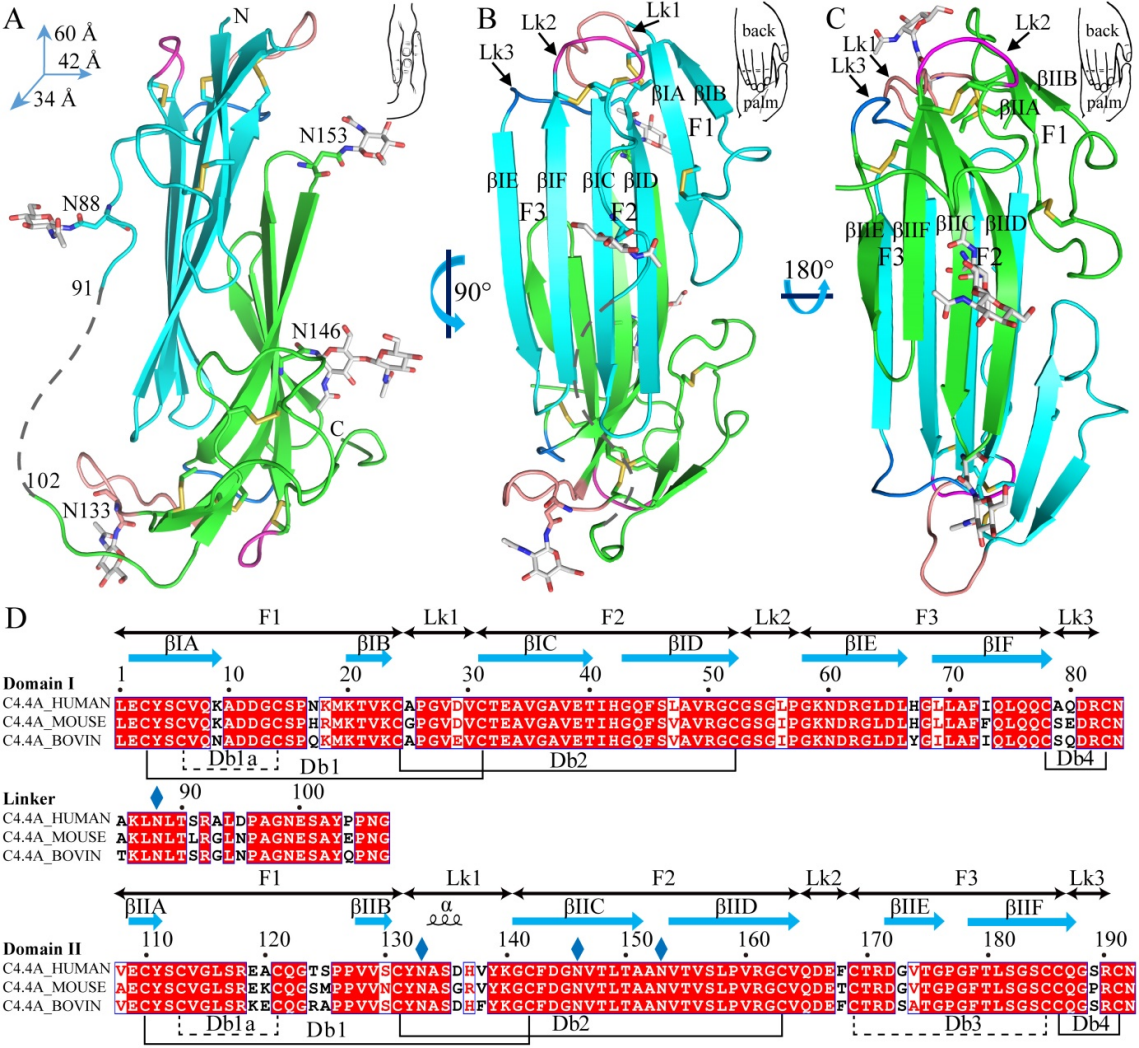
The crystal structure of C4.4A and sequence alignment of different C4.4A. **(A, B and C)** Three perspective of the structure of C4.4A. The schematic drawings using two hands to show the relative orientation of the two domains. Both DI (cyan, residues 1-91) (B) and DII (green, residues 102-201) **(C)** contain similar six β-strands (βA to βF) and three linkers (Lk1 in color salmon, Lk2 in blue and Lk3 in purple). These β-strands constitute three fingers. The N-linked glycosylation are highlighted as grey sticks. The disulfide bonds are shown as yellow sticks. All structural figures here and below are prepared by PyMol 64. **(D)** Sequence alignments of the C4.4A from different species. Strictly conserved amino acids are show in red background while strictly conserved disulfide bonds (Db1, Db2 and Db4) in solid line and highly conserved disulfide bonds (Db1a and Db3) in dash lines. The α-helix and β-strand of secondary structure are shown above the sequence as spiral curve and arrow, respectively. Diamonds denote potential N-linked glycosylation sites. The sequence alignment was made by ESPript 3.0 65.

The current crystal structure (Fig. 1) shows that each LU-domain (DI, residues 1-91, DII, residues 102-201) in C4.4A contains six β-strands (DI: βIA, regions 2-9; βIB, 20-23; βIC, 31-40; βID, 43-52; βIE, 58-66; βF6, 69-78; DII: βIIA, 109-111; βIIB, 128-130; βIIC, 141-151; βIID, 154-164; βIIE, 172-176; βIIF, 179-187) providing a scaffold for the assembly of three protruding loops (also named fingers: F1, βA to βB; F2, βC to βD; F3, βE to βF)—one of the topological hallmarks defining the archetypical three-fingered fold 4, 28. At the disulfide-rich base (“palm region”), three linker regions (Lk1, Lk2 and Lk3) join the individual loops (*i.e.*, F1 to F2 by connecting βB-βC, F2 to F3 by connecting βD-βE, and F3 to the C-terminal region *via* βF). Notably, the DII-Lk1 connecting βIIB and βIIC of C4.4A is quite long (9 amino acids *vs* only 6 in the DI-Lk1). The six β-strands in the DI form a large continuous β-sheet (61% β-sheet content), whereas the DII has a small β-sheet (49% β-sheet content) (Fig. 1D).

A long inter-domain linker exists between the two LU-domains in C4.4A. However, the two LU-domains assemble *via* a large hydrophobic interface to form a compact protein structure with the dimensions 60 x 42 x 34 Å (Fig. 1A, B and C). This unique assembly of the LU-domains in C4.4A resembles two right hands tightly facing each other on finger area.

There are two C4.4A molecules in the asymmetric unit of the crystal. Superposition of the two molecules shows that their structures are highly similar with each other with RMSD of 0.72 Å for all Cα, further supporting the low flexibility of the structure under these conditions. Another notable key difference between the two LU-domains in C4.4A is the arrangement and the number of disulfide bonds. The DI has four disulfide bonds (Db1, Cys3-Cys31; Db1a, Cys6-Cys14; Db2, Cys24-Cys52; Db4, Cys78-Cys83), while the DII has five (Db1, Cys110-Cys142; Db1a, Cys113-Cys121; Db2, Cys131-Cys163; Db3, Cys169-Cys185; Db4, Cys186-Cys191) (Fig. 1D). This divergent arrangement of the disulfide bonding is nonetheless not unique to C4.4A, but is found in all proteins with multiple LU-domains. In these proteins, the N-terminal LU-domains invariably lack one of the otherwise consensus disulfide bonds (Db3) 2. Paradoxically, missense mutations affecting one of the four consensus disulfides in the *single* LU-domain-containing proteins (*e.g.*, GPIHBP1, CD59, κ-bungarotoxin) cause protein misfolding and loss-of-function 6, 12, 62, 63. One possible structural advantage of the absence of Db3 in the DI is that the affected βID becomes much less twisted compared to the corresponding βIID in the DII (where Db3 remains intact). A comparison to all structures solved for uPAR reveals similar lower twisting of βID compared to βIID 28-32.

The crystal structure revealed clearly four N-linked glycans (Fig. 1A, D), including one located at the linker regions between the LU-domains of C4.4A (Asn88) and three glycans in the DII of C4.4A (Asn133, Asn146 and Asn153).

### Structural basis for a compact conformation of C4.4A

**Figure 2:**
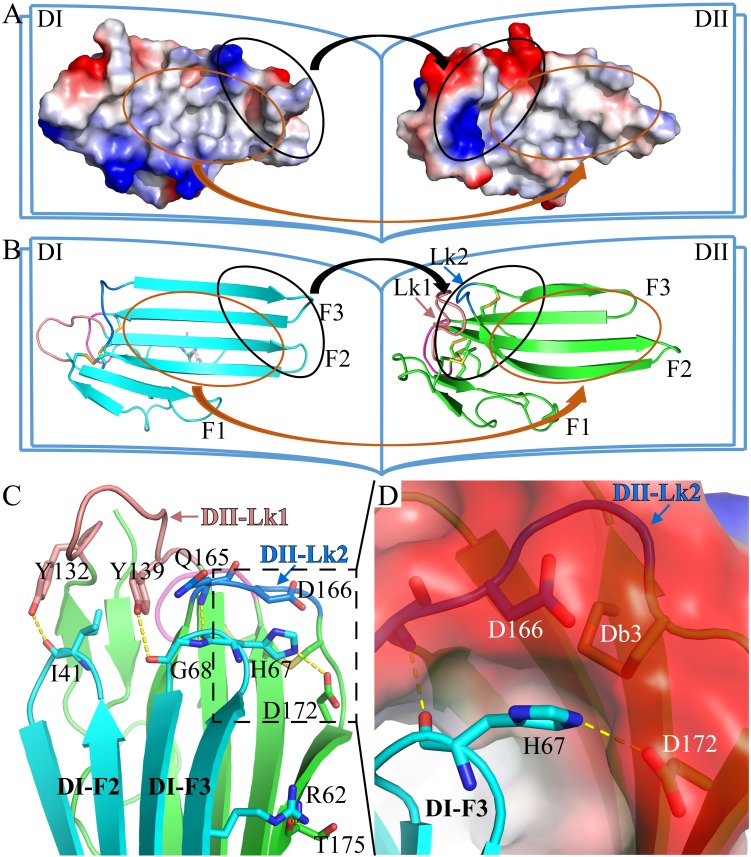
The crystal structure of C4.4A. Open-book illustration of the two C4.4A domains shown in electrostatic surface **(A)** and cartoon **(B)** representations. The interaction between the two LU-domains is mediated mainly by hydrophobic force (orange circle) together by some polar interaction (black circle). Two LU-domains have high complementarity in shape. Blue indicates positive potential and red indicates negative potential. The polar interaction between two domains of C4.4A is mainly located in the palm of DII and the tip of fingers (F2 and F3) of DI. The connecting peptide between βIID and βIIE in DII (Lk2, blue arrow) is twisted toward its central 3-stranded β-sheet. The Lk1 (orange arrow) connecting βIIB and βIIC adopts a similar orientation. **(C)** Polar interaction (yellow dashed lines: Ile41 to Tyr132; His67 to Gln165; His67 to Asp172; Gly68 to Tyr132; Arg62 to Thr175) contribute to the formation of interaction between the two LU-domains. **(D)** A negatively charged pocket in DII (harboring Asp166 and Asp172 and stabilized by Db3) is occupied by His67 in the tip of DI-F2.

The two LU-domains of C4.4A associate tightly in the crystal structure forming a globular protein. This domain organization is predominantly stabilized *via* interdomain hydrophobic interactions involving relatively large surfaces of the central β-sheet in each LU-domain (Fig. 2A, B). Of note, the central β-sheets of the two LU-domains in C4.4A are both asymmetric in the sense that they have one face which is particularly hydrophobic (hydrophobic contact area of 562 Å^2^ for DI and 638.6 Å^2^ for DII) (Table S1). These hydrophobic faces of the β-sheets assemble to form the interdomain binding interface and they share a high degree of shape complementarity. A number of polar interactions are also found at the rim of the interdomain interface: Ile41-Tyr132, Arg62-Thr175, His67-Gln165, His67-Asp172, Gly68-Tyr139 (Table S2, Fig. 2C). These hydrogen bonds and ionic interactions likely provide a directional force to stabilize the relative orientation between two LU-domains. Interestingly, these polar interactions are predominantly located at the interface created by the shape complementarity between the finger tips in the DI (finger tips of F2 and F3) and the disulfide rich core of the DII (including Lk1 and Lk2). In this region, the DII forms a highly negatively charged pocket (Asp166 and Asp172) that accommodates His67 from the DI by electrostatic interaction (Fig. 2D). This pocket is stabilized by Db3.

### Antibody 11H10 recognized both LU-domains in C4.4A

**Figure 3:**
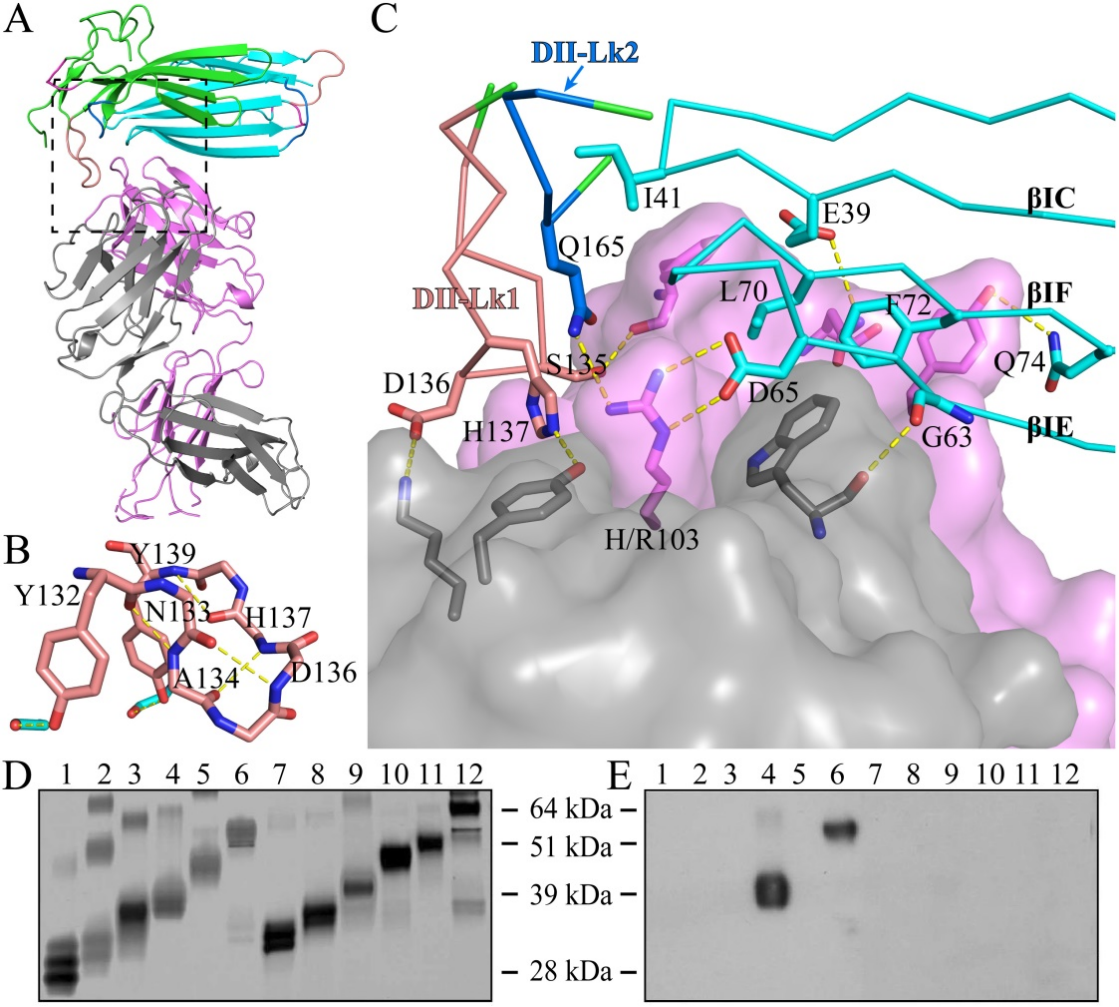
The crystal structure of C4.4A:Fab complex. **(A)** The mouse monoclonal anti-human C4.4A antibody (11H10) recognizes a composite epitope on human C4.4A that is assembled by the strands of DI (cyan) with the linker between strands of DII (green). The light chain and heavy chain of antibody are colored gray and violet, respectively. **(B)** The internal hydrogen bond network in both DII-Lk1 of C4.4A and C4.4A:Fab complex. All residues, except Tyr132 and Tyr139, are shown in sticks only main chain atoms. The DI residues are colored in cyan. **(C)** The details of interaction between C4.4A and Fab shows that Fab fragment (shown in surface with a 60% transparency) is get caught by C4.4A (shown in ribbon) thought three β-strands (βIC, βIE and βIF) of DI and the linkers between β-strands of DII (Lk1 and Lk2). The key residues of C4.4A are shown in sticks and labeled. **(D and E)** Mapping domain reactivity of mAb 11H10 with various C4.4A fragments constructs. **(D)** Silver stained SDS-PAGE gel of purified C4.4A domain constructs fused to a C-terminal uPAR DIII purification tag: C4.4A-DI^1-107^ (lanes 1 and 7), C4.4A-DII^108-199^ (lanes 2 and 8), C4.4A-STP^200-278^ (lanes 3 and 9), C4.4A-DIDII^1-199^ (lanes 4 and 10), C4.4A-DIISTP^108-278^ (lanes 5 and 11), and C4.4A^1-278^ (lanes 6 and 12). Samples in lane 1-6 were unreduced, while samples in lane 7-12 were reduced and alkylated prior to SDS-PAGE. **(E)** An identical set of samples were immobilized on a PVDF-membrane. After blocking excess of binding sites, the PVDF membrane was incubated with 1 µg/mL of mAb 11H10 as primary antibody and was visualized by enhanced chemiluminescence. The results show mAb 11H10 only reacts with constructs containing both LU-domains in C4.4A and only if the disulfide bonds remained intact (lanes 4 and 6).

The structure of C4.4A in the complex with the Fab fragment of mAb 11H10 is highly similar to the C4.4A alone (Fig. 3A), with an RMSD of 0.55 Å for all atoms. This high similarity demonstrates the compactness and rigidity of the globular assembly of the two LU-domains in C4.4A is not affected by the crystal lattice formation and the presence of the antibody. Note that the complex was crystallized under neutral pH (7.0), compared to the low pH (3.6) crystallization of C4.4A, which further underlines the stability of the compact structure of C4.4A.

As shown by the C4.4A:Fab complex structures, the structural epitope on C4.4A recognized by the Fab fragment is mainly located in three β-strands (βC, βE and βF) in DI and the linkers between β-strands in DII (Lk1 and Lk2) (Fig. 3C, Table S3). The Fab Arg103 of heavy chain (*labeled as H/Arg103 in Fig. 3C*) inserts into the groove of C4.4A-DI and C4.4A-DII and forms hydrogen bonds with Asp65 and Gln165 of C4.4A (Table S2). On the other hand, C4.4A-DI residues Leu70 and Phe72 embed into the hydrophobic area surrounded by Fab heavy chain (Phe32, Trp54, Trp55, Tyr58, Tyr60 and Leu102) and light chain (Trp94 and Pro95).

Usually, the conformation of loop is susceptible to ligand binding and/or environment due to its flexibility. However, despite containing a long loop in DII-Lk1, the binding of Fab doesn't induce the conformational change of this loop. Further structural analysis shows Tyr132 and Tyr139 located in DII-Lk1 form hydrogen bonds with DI to stable the conformation of DII-Lk1 (Fig. 3B). Moreover, the DII-Lk1 appears to have constrained conformation due to the presence of four internal hydrogen bonds (Tyr132-Ala134, Asn133-Asp136, Ala134-His137, His137-Tyr139). All hydrogen bonds are mediated by the main chain atoms and thus are conserved in different species.

The structure of the complex demonstrates that the mAb 11H10 recognizes a conformational epitope on intact C4.4A-DIDII containing both LU-domains. This observation is excellently aligned with biochemical results showing by Western Blot (Fig. 3D, E) that the binding of mAb 11H10 to C4.4A requires that both domains are present and that it is folded correctly (*line 4 and 6*).

## Discussion

### The functional site of C4.4A for ligand binding

C4.4A was reported to interact with both α6β4 integrin and MMP14, promoting wound healing and metastasis 45. In addition, the interaction between C4.4A with Anterior Gradient 2 (AGR2) stimulates pancreatic ductal adenocarcinoma cell aggressiveness and reduces sensitivity to chemotherapy drug gemcitabine. C4.4A also binds to integrin β1 and laminins 1 and 5 46. However, the structural details of how C4.4A interacts with its ligands is unknown.

**Figure 4:**
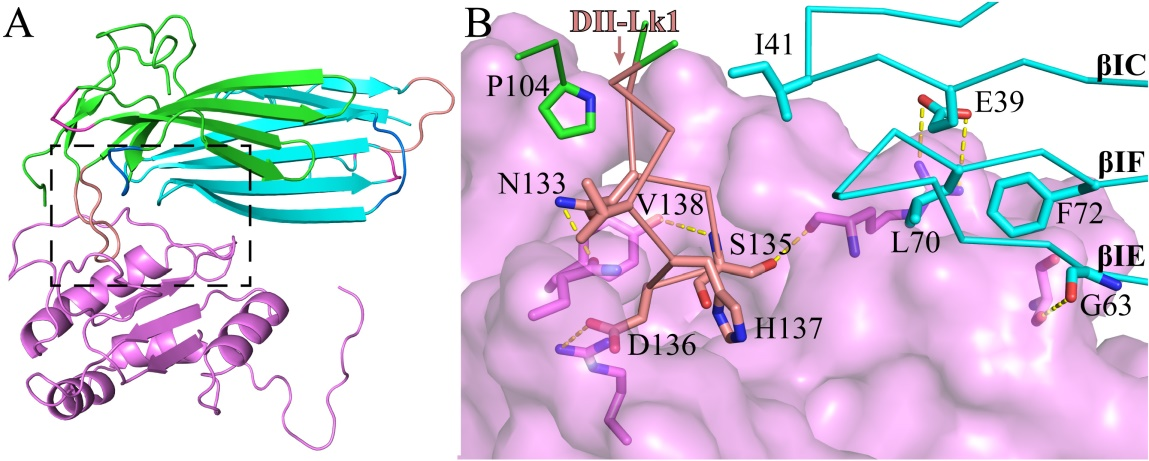
The molecular model of C4.4A (cyan & green) complex with AGR2 (violet). **(A)** Overall structure of C4.4A:AGR2 is quite similar to C4.4A:Fab (Fig. 3A). **(B)** The details of interaction between C4.4A and AGR2. The DII-Lk1 of C4.4A inserts into the hydrophilic pocket of AGR2 (violet surface and sticks). The βIC, βIF, and βIE strands of DI also interact with AGR2.

Based on our crystal structure of C4.4A:Fab complex, we studied its molecular interaction of C4.4A with AGR2 by carrying out the protein-protein docking between C4.4A and AGR2 (PDB ID: 2lnt) 66 using ZDOCK (Version 3.0.2) 67. The top docking solution clearly stands out from all the rest of the solutions, demonstrating the top solution is highly reliable. Interestingly, the AGR2 contacts to C4.4A at the site (Fig. 4A) quite close to the Fab fragment binding site (Fig. 3A). Again, the C4.4A DII-Lk1 moiety plays an important role mediating the interaction by docking into a pocket of AGR2 (Fig. 4B). These consistent results demonstrate that this area of C4.4A is important for ligand binding.

### A novel assembly mode of LU-domains in C4.4A

LU-domains contain three to six highly conserved disulfide bonds with a unique signature motif: CCxxxxCN (x is arbitrary amino acid), which is tightly packed at the palm region 68.

**Figure 5:**
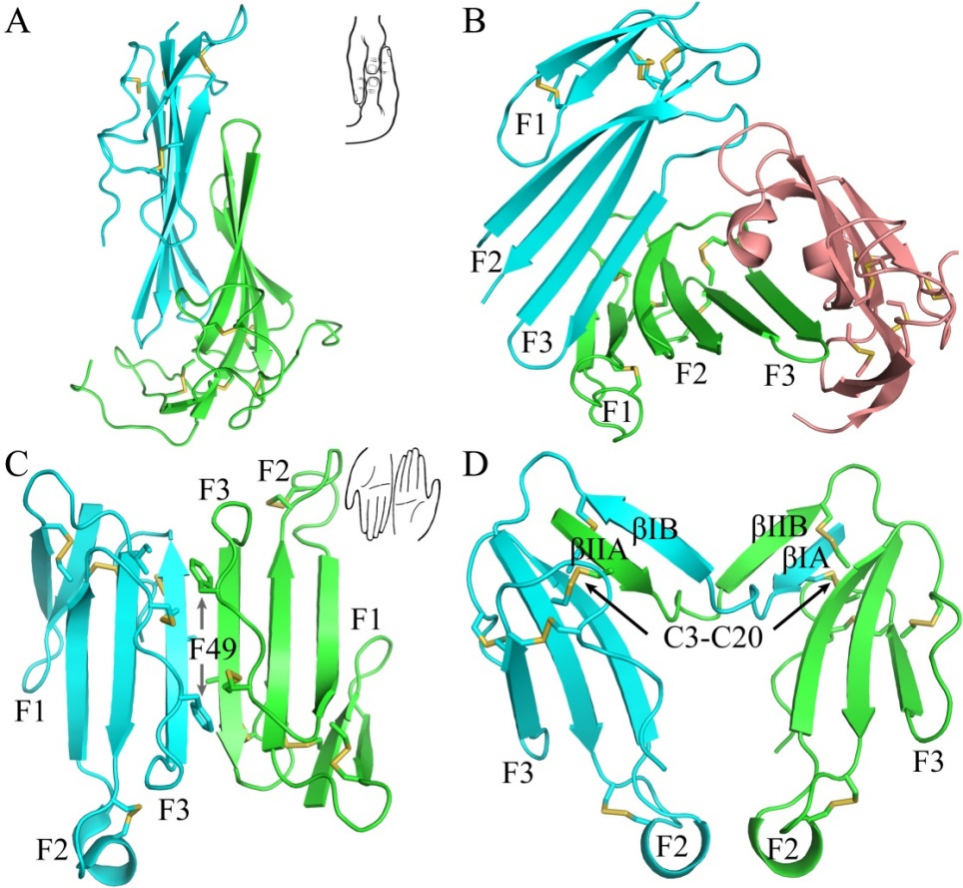
Different ways of LU-domain association. The assemble mode in crystal structures of **(A)** C4.4A (PDB ID: 6IOM), **(B)** uPAR (PDB ID: 2FD6), **(C)** β-Bungarotoxin homodimer (PDB ID: 1KBA) and **(D)** α-cobratoxin homodimer (PDB ID: 4AEA). Selected involved in interaction side chains were shown in sticks. Disulfide bonds were shown in yellow sticks.

In many cases, the palm surface of LU-domain is important in interacting with the ligand, as shown by the structure of the multiple-LU-domains protein (uPAR) and single LU-domain protein (CD59 and some three-fingered snake venom toxins) 29, 69-71. The uPAR contains three LU-domains, which assemble in a circular manner by interdigitating with each other to generate a central cavity (Fig. 5B) which accommodate its ligand. However, in our C4.4A structures, the palm surfaces of two domains are composed wholly of hydrophobic residues and buried inside the protein by the unique face-to-face assembly mode of two LU-domains.

A novel mode of homodimerization of LU-domains was revealed in our C4.4A structure. All of known three-fingered snake venom toxins contains only one LU-domain. A few toxins exist nevertheless as non-covalent homodimers in solution e.g. κ-bungarotoxin and haditoxin 11, 72. In these dimers, two independent protein molecules are arranged in an antiparallel manner (Fig. 5C). The interaction between the protomers consist of the pairing of β-strands and van der Waals interactions provided by some hydrophobic residues in the F3. The key residue Phe49, which is found in all four κ-bungarotoxins to provide the hydrophobic core, interact with Ile20, Thr60 and the disulfide bond Cys46-Cys58 from another subunit 12, 73. Three-fingered snake venom toxins also form homodimers or heterodimers *via* intermolecular disulfide bonds 74. In the α-cobratoxin homodimer, the first N-terminal β-strand of two protomers were swapped, and two intermolecular disulfide bonds were formed between Cys3 in one protomer and Cys20 in another (Fig. 5D) 13.

### Prediction of structure of Haldisin, a C4.4A analogue, based on C4.4A structure

**Figure 6:**
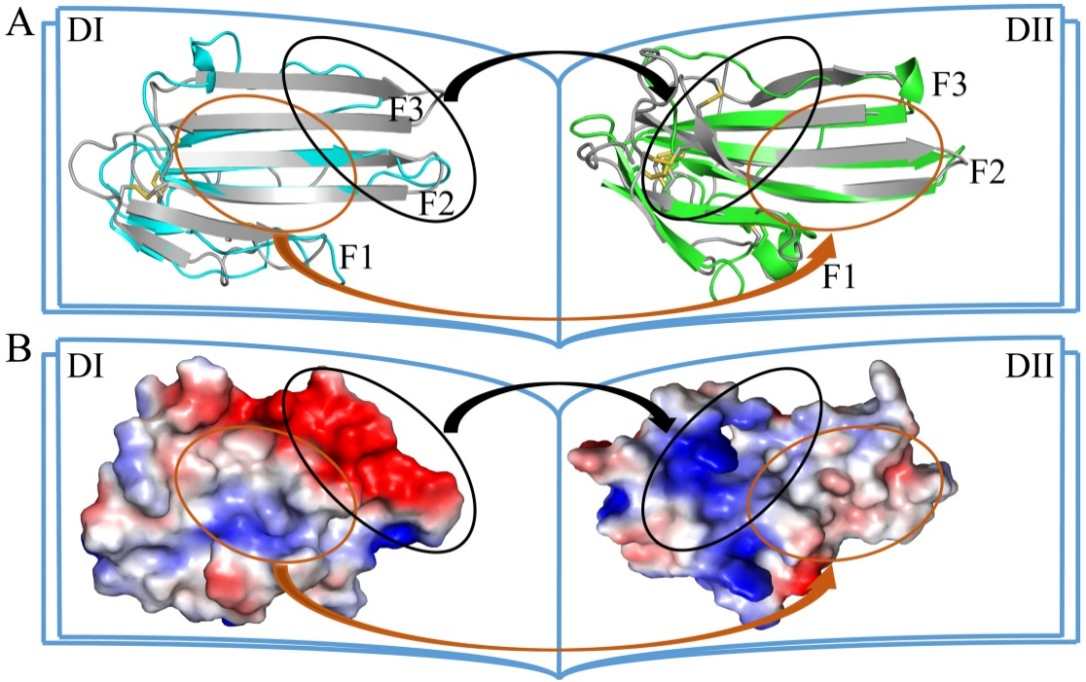
The structural model of Haldisin and C4.4A. **(A)** The superposition of Haldisin (DI colored in cyan, DII colored in green) and C4.4A (grey) shows that two structures fit well except some deviation of βIIE of DII. The inter-domain assembly of Haldisin is similar to C4.4A. **(B)** shows the surface electrostatic potential of the LU-domains the same orientation as in (A).

Haldisin (encoded by *LYPD5*) is extracellular protein predominantly expressed in stratum granulosum of human skin under homeostatic condition, and was predicted to contain two LU-domains with disulfide bonding pattern similar to C4.4A 25. However, the sequence identity between Haldisin and C4.4A is low, particularly for the DI (Fig. S1). Despite this low sequence conservation, we were able to generate a homology model of Haldisin based on our structure of C4.4A. The model was subjected to thorough molecular dynamics (MD) simulation. The stability, sampling and convergence of the MD simulation were established by calculation of the backbone RMSD (Fig. S2). Hess analysis and RMSD both confirmed the adequate sampling of Haldisin conformation around the equilibrium position in the last 500 ns of the MD simulation (Table S5). The most representative model of Haldisin, covering 92% of the sampled conformations was identified by clustering analysis on the last 500 ns MD trajectory. The resultant Haldisin model showed high structural similarity to our crystal structure of C4.4A with the RMSD of 1.58 Å (DI) and 2.16 Å (DII) for all Cα atoms (Fig. 6A). Importantly, the inter-domain interface of Haldisin is highly complementary to each other in term of charges (Fig. 6B blank circles) and polarity (Fig. 6B orange circles). Such high degree of structural similarity of Haldisin to C4.4A suggests parallel functions, which remains to be confirmed experimentally.

## Experimental procedures

### Generation of a monoclonal anti-C4.4A antibody and its Fab fragment

A mouse monoclonal anti-C4.4A antibody (11H10) was generated by conventional mouse hybridoma technology after immunizing FVB mice with purified recombinant human C4.4A produced in *Drosophila* S2-cells with a C-terminal uPAR DIII fusion tag that was removed by enterokinase treatment 56, 75. Purified 11H10 was treated with immobilized Ficin (Thermo Scientific, Rockford, Il, US) in the presence of 25 mM L-cysteine and 1 mM EDTA at 37°C for 1 h to produce Fab and Fc fragments. The reaction mixture was passed through a Protein A column to remove the undigested 11H10 and its Fc fragments. The Fab-containing flow-through fraction was further purified size exclusion chromatography using a Superdex 200.

### Sequencing the CDRs of mouse monoclonal anti-C4.4A antibody 11H10

Hybridomas producing 11H10 mAb (IgG1κ) were cultured and used to generate cDNA for the corresponding light and heavy chains using the Cells-to-cDNA^TM^ II kit (Life Technologies) and the following primers:

Vκ light-chain 5`-primer:

TATGAATTCGACATTCTGATGACCCAGTCT;

Cκ light-chain 3`-primer:

AGCGGCCGCACACTCATTCCTGTTGAAGCTCTTGAC;

V_H_ heavy-chain 5`-primer:

TATGAATTCCAGGTTACTCTGAAAGAGTCTGG;

C_H_ heavy-chain 3`-primer:

AGCGGCCGCACAATCCCTGGGCACAATTTTCTTGTC.

The cDNAs were amplified with conventional PCR using the Platinum Pfx DNA Polymerase (Invitrogen) and products with the proper size (app. 700 bp) were purified from a 1% agarose gel with the QiaQuick Gel Extraction Kit (Qiagen). The cDNAs were cloned into pBlueScript KS+ using the introduced EcoRI and NotI restriction enzyme sites (underlined in the primer sequences) and the Rapid DNA ligation kit (Roche). Subsequently, DH5α Competent cells were transformed and DNA was isolated from individual clones and analyzed by restriction enzyme digestion before sequencing. Five-six clones for each chain were sequenced and revealed 100% identical sequences. The amino acid sequences are shown below:

11H10 Fab light chain:

DILMTQSPAILSVSPGEGVSFSCWANQNIGTSIHWYQQRTNGSPRLLIKYASESISGIPSRFSGSGSGTDFTLSINSVESEDIADYYCQQSNSWPIFTFGSGTKLEIKRADAAPTVSIFPPSSEQLTSGGASVVCFLNNFYPKDINVKWKIDGSERQNGVLNSWTDQDSKDSTYSMSSTLTLTKDEYERHNSYTCEATHKTSTSPIVKSFNRNEC.

11H10 Fab heavy chain:

QVTLKESGPGILQPSQTLSLTCSFSGFSLNSFGTGVGWIRQPSGKGLEWLAHIWWNDYKYYNAALESRLTISKDTSNNQVFLKIASVDTADTATYYCARLRLRYFDYWGQGTTLTVSSAKTTPPSVYPLAPGSAAQTNSMVTLGCLVKGYFPEPVTVTWNSGSLSSGVHTFPAVLQSDLYTLSSSVTVPSSTWPSETVTCNVAHPASSTKVDKKIVPRDC.

### Expression and purification of soluble human C4.4A

Recombinant C4.4A-ent-uPAR-DIII fusion protein was produced in *Drosophila* S2 cells and purified by immunoaffinity chromatography as described 59, 75. The C4.4A protein containing the two LU-domains was subjected to limited proteolysis with chymotrypsin preferential hydrolyzing the peptide bonds after Tyr200 or Phe201 in the linker region between the DII and the mucin-type C-terminal domain 42 and further purified by size exclusion chromatography using a Superdex75 column 60.

### Immunoblotting Analysis

The generation of various C4.4A domain constructs was produced in *Drosophila* S2 cells and purified by immunoaffinity chromatography as described 59. After separation by SDS/PAGE an identical set of samples were immobilized on a PVDF-membrane (Millipore, Bedford, MA, U.S.A.). After blocking excess of binding sites, the PVDF membrane was incubated with 1 µg/ml of mAb 11H10 as primary antibody and peroxidase conjugated swine anti-mouse immunoglobulins (Dako, Glostrup, Denmark) diluted 1:5000 as secondary antibody. Positive reactivity was visualized by enhanced chemiluminescence (ECL plus; Amersham).

### Formation of complex between C4.4A and 11H10 Fab

The Fab peak was pooled and mixed with C4.4A-DIDII and the resultant complexes purified by gel filtration on a Superdex 200 column (GE Life Sciences) with 20 mM Tris, 150 mM NaCl and pH 7.4 as the running buffer. The eluted fragments contained the target complex were mixed and concentrated to 10 mg/mL using an Amicon Ultra Centrifugal Filter Device (Millipore, USA) with a molecular mass cutoff of 10,000 Da, the aliquots were stored frozen at -80°C.

### Crystallization

The C4.4A crystals were obtained at 293K by the sitting-drop vapor-diffusion method in a concentration of 10 mg/mL. The precipitant condition is 22.5% (*w/v*) polyethylene glycol 4000, 0.1 *M* citric acid in pH 3.6, as described previously 60. For C4.4A:Fab complex, all crystallization trials were done at 295K using commercial screening kits (from Qiagen, XtalQuest and Hampton Research) with the Phoenix robot (Art Robbins Instruments). Optimized crystals grew in 20% PEG3350, 0.2M Potassium sodium tartrate tetrahydrate, 0.1M Tris-HCl pH 7. Crystals appeared within the third day and grew larger over the course of 2 weeks. Slender rod-shaped crystals were carefully looped and frozen in ice-free liquid nitrogen after a quick soak in original mother liquor with 25% (v/v) glycol.

### Data collection and processing

Prior to X-ray data collection, the crystals were transferred to the precipitant solution containing 25% (*v/v*) glycerol and flash-frozen in liquid nitrogen. Diffraction data for both C4.4A and C4.4A:Fab complex were collected on beamline X29 at Brookhaven National Synchrotron Light Source (NSLS) and were processed using the automated data-processing pipeline xia2 76 with options that run XDS 77.

### Structural determination of C4.4A-Fab complex and C4.4A

MR method was used to solve C4.4A:Fab structure. The Fab fragment was positioned into the crystal lattice using *BALBES* automated structure-solution pipeline. Model refinement and building were carried out in PHENIX and COOT reiterally until converged. Next, search models of C4.4-DIDII were prepared by Sculptor utility of the PHENIX suite to match the target sequence based on the published human suPAR structure (PDB 3BT2, chain U, the DI and DII of C4.4A share 30% and 28% sequence identity with suPAR DII respectively). No MR solution can be found with all these models alone. Importantly, with the Fab positioned, we obtained the MR solution of the model of DI, giving a *z*-score of translational function higher than 12.0. The log-likelihood gain (LLG) from Phaser was also positive, indicative of a successful MR solution. The model was adjusted according to electron density map in Coot.

After several rounds of refinement of the DI in C4.4A:Fab crystal, the well-fit model of DI was used as the search model for C4.4A crystal dataset, which had higher resolution (2.6 Å) than C4.4A:Fab complex (2.8 Å). The MR using Phaser produced the successful solution, and identified both molecules in the asymmetric unit. Phenix Autobuild module was used to perform iterative model building, refinement and density modification, leading to an improved electron density map. Iterative cycles of model building and refinement were performed until the model cannot be improved.

The model from the C4.4A crystal was then used as the searching model to successfully place into the C4.4A:Fab crystal by MR method with the positioned Fab as fixed model. The completed model of the complex was further improved by several cycles of refinement and manual adjustment until converged. All relevant data collection and refinement statistics were summarized in Table 1.

### Molecular dynamics simulation of Haldisin

The homology model of Haldisin was built automatically by the SWISSmodel web server based on the crystal structure of C4.4A 78. The model of Haldisin was inserted into a truncated octahedron water box with edge lengths of 71 Å, 71 Å, and 71 Å. The protonation states of residues were assigned according to the corresponding p*K*_a_ values calculated by using the H++ webserver 79. Two Na^+^ ions were added to counterbalance the charge of the protein. The system contained 8,630 water molecules and 28,786 atoms in total. It was underwent MD simulations with AMBER ff99SB-ILDN force field 80-82 using the GROMACS 4.6.5 code 83. The Åqvist potential 84 and TIP3P model 85 were used for the ions and for the water molecules, respectively. All bond lengths were constrained by LINCS algorithm 86. Periodic boundary conditions were applied. Electrostatic interactions were calculated using the Particle Mesh-Ewald (PME) method 87, and van der Waals and Coulomb interactions were truncated at 10 Å. The system underwent 1,000 steps of steepest-descent energy minimization with 1,000 kJ·mol^-1^·Å^-2^ harmonic position restraints on the protein, followed by 2,500 steps of steepest-descent and 2,500 steps of conjugate-gradient minimization without restraints. The system was then gradually heated from 0 K up to 298 K in 20 steps of 2 ns. After that, 2000 ns-long productive MD simulations were carried out in the NPT ensemble. The most representative structure was identified by the cluster analysis 83 with a cut-off of 1.5 Å over the equilibrated trajectories, ranging from 1500 ns to 2000 ns. To assess the convergence of the simulated trajectory in the last 500 ns, we considered the projection of each snapshot on the top essential dynamical spaces obtained from a standard covariance analysis. Following Hess's criterion 88, these projections were next compared with those expected for a random reference. The observed negligible overlap (i.e. cosine content close to 0, see Table S5) confirmed a posterior adequate sampling of Haldisin conformation around the equilibrium position in the last 500 ns.

## Supplementary Material

Supplementary figures and tables.

## Figures and Tables

**Table 1:** Data collection and structure refinement statistics.

	C4.4A	C4.4A:Fab
*Data Collection and scaling*		
Resolution range	49.309 - 2.59 (2.69 - 2.59)	40.144 - 2.75 (2.85 - 2.75)
Space group	C 2 2 2_1_	P 1 2_1_ 1
*Cell dimensions*		
a, b, c (Å)	57.1, 120.2, 169.4	51.9, 64.0, 107.7
α, β, γ (º)	90, 90, 90	90, 96.1, 90
Unique reflections	18403 (1763)	18267 (1809)
Redundancy	7.7 (8.1)	3.7 (3.8)
Completeness (%)	99.49 (97.18)	98.91 (99.07)
*I*/σ(*I*)	37.58 (3.76)	8.90 (2.09)
Wilson B-factor	55.43	54.15
R-merge^a^	0.094 (0.690)	0.1134 (0.8406)
*Refinement*		
R-work	0.2063 (0.3126)	0.1963 (0.3068)
R-free	0.2503 (0.3349)	0.2555 (0.3484)
Average B-factor (Å^2^)	70.46	77.20
Macromolecules	69.53	77.04
ligands	96.99	90.27
*Validation*		
RMSD from ideal		
Bond lengths (Å)	0.011	0.013
Bond angles (°)	1.63	1.88
Ramachandran plot		
favoured (%)	92.80	95.30
outliers (%)	0.00	0.50
PDB code	6IOM	6ION

Statistics for the highest-resolution shell are shown in parentheses. ^a^Rmerge=Σ|*I*_i_-<I>|/Σ*I*_i,_ where *I_i_*is the intensity of the *i*th observation and <*I*> is the mean intensity of the reflections.

## Abbreviations

LU-domains: Ly6/uPAR/α-neurotoxin domains
GPI: glycosyl-phosphatidylinositol
uPAR: urokinase-type plasminogen activator receptor
DI: the first domain
DII: the second domain
DIII: the third domain
MR: molecular replacement
STP-rich region: mucin-type region rich in serine, threonine, and proline
AGR2: Anterior Gradient 2
MD: molecular dynamics
