# Supplementary material for: Crystal Structures of Human C4.4A Reveal the Unique Association of Ly6/uPAR/α-neurotoxin Domain
Source: Int J Biol Sci. 2020 Jan 30;16(6):981–93. doi: 10.7150/ijbs.39919 (PMC7053344; doi:10.7150/ijbs.39919)
Supplement: Supplementary file 1 — Supplementary figures and tables. [file ijbsv16p0981s1.pdf]

Supplemental Material for

Crystal Structures of Human C4.4A Reveal the Unique Association of Ly6/uPAR/α-neurotoxin Domain

Yunbin Jiang[1,2], Lin Lin[3*], Shanli Chen[4], Longguang Jiang[4], Mette C. Kriegbaum[5,6], Henrik Gårdsvoll[5,6], Line V. Hansen[5], Jinyu Li[4], Michael Ploug[5,6], Cai Yuan[7*], Mingdong Huang[1,4*]

[1]State Key Laboratory of Structure Chemistry, Fujian Institute of Research on the Structure of Matter, Chinese Academy of Sciences, Fuzhou, Fujian, 350002, China;

[2]University of Chinese Academy of Sciences, Beijing 100049, China;

[3]Institute of Oceanography, Minjiang University, Fuzhou, 350108, China;

[4]College of Chemistry, Fuzhou University, Fuzhou, Fujian, China;

[5]Finsen Laboratory, Rigshospitalet, DK-2200 Copenhagen N, Denmark;

[6]Biotech Research and Innovation Centre (BRIC), University of Copenhagen, DK-2220 Copenhagen N, Denmark;

[7]College of Biological Science and Engineering, Fuzhou University, Fuzhou, Fujian, 350116, China;

***Corresponding author**: Mingdong Huang, Tel.: +86-13859021285, E-mail: HMD_lab@fzu.edu.cn; Cai Yuan, Tel.: +86-13599062093, E-mail: cyuan@fzu.edu.cn; Lin Lin, E-mail: llinlynn2@163.com;

Keywords: C4.4A, uPAR, three-fingered fold, LU-domain;

Table S1: The residues and their contact area of the interaction of two LU-domains.

Residue/Chain A	Contact Area/Å^2	Residue/Chain B	Contact Area/Å^2
I41	69.27	V156	49.18
L66	55.61	V114	49.00
F45	50.64	L149	47.16
I73	48.15	P177	44.53
L47	45.30	L158	42.25
V49	41.07	F179	41.01
L69	40.31	Y132	36.93
K9	34.48	Y139	33.18
L64	30.75	V174	28.58
V38	18.33	L181	27.01
A34	17.98	A152	25.47
A71	15.22	V147	25.26
T40	14.97	V164	22.68
V7	12.43	F143	22.06
A10	11.11	A151	20.77
G51	9.28	C163	18.73
L75	8.08	V160	17.71
G43	7.33	V129	16.78
G36	7.01	V154	12.74
Q44	6.80	G115	11.52
T32	4.97	C169	10.03
A48	4.63	G173	9.61
D65	4.33	A134	7.15
Q8	1.90	S157	6.33
R50	1.24	C185	3.71
V35	0.93	G176	3.37
		C131	2.17
		T148	1.55
		S182	1.24
		P159	0.95

Table S2: Hydrogen bonds in the interface of C4.4A and C4.4A:Fab

C4.4A-DI	C4.4A-DII	[a]Fab	Distance (Å)
Ile^{41} (O)	Tyr^{132} (OH)	-	2.48
Arg^{62} (NH2)	Thr^{175} (O)	-	2.92
His^{67} (O)	Gln^{165} (N)	-	2.82
His^{67} (NE2)	Asp^{172} (OD2)	-	2.83
Gly^{68} (O)	Tyr^{139} (OH)	-	3.35
Glu^{39} (OE2)	-	H/Asn^{56}(ND2)	4.00
Gly^{63} (O)	-	L/Trp^{94}(O)	3.28
Asp^{65} (OD1)	-	H/Arg^{103} (NH2)	2.78
Asp^{65} (OD2)	-	H/Arg^{103} (NE)	2.78
Gln^{74} (NE2)	-	H/Tyr^{58} (OH)	3.51
-	Ser^{135} (OG)	H/Phe^{32} (O)	2.70
-	Asp^{136} (OD2)	L/Lys^{49} (NZ)	3.12
-	His^{137} (NE2)	L/Tyr^{50} (OH)	2.51
-	Gln^{165} (NE2)	H/Arg^{103} (NH1)	3.44

[a]Fab: H means heavy chain and L means light chain.

Table S3: The residues and their contact area of the interaction between C4.4A and Fab fragment.

Residue/C4.4A	Contact Area/Å^2	Residue/[a]Fab	Contact Area/Å^2
F72	60.89	H/F32	81.14
H137	44.44	L/W94	51.01
S135	36.92	H/Y58	45.44
E39	36.27	H/R103	39.52
G63	33.89	H/Y60	33.74
L70	30.13	H/R101	24.68
D65	26.33	H/L102	19.57
I41	18.85	H/N56	17.71
Q74	14.71	H/W55	17.40
Q165	12.83	L/P95	13.05
D136	12.08	L/Y50	12.82
G68	11.47	H/Y104	8.99
L64	9.98	L/K49	8.35
Y139	9.18	H/G33	6.52
R62	8.18	H/W54	5.28
T40	8.17	H/T34	2.95
A37	7.77		
A134	5.28		

[a]Fab: H means heavy chain and L means light chain.

Table S4: The residues and their contact area of the interaction between C4.4A and AGR2.

Residue/C4.4A	Contact Area/Å^2	Residue/AGR2	Contact Area/Å^2
D136	61.01	Q123	74.52
P104	53.86	A139	71.62
F72	52.50	R138	43.09
Y132	50.00	S134	42.95
L70	48.46	T136	41.43
S135	44.77	Y150	41.29
H137	34.19	T142	32.92
N133	32.57	V137	25.37
A134	24.05	I141	24.15
V138	23.75	M129	23.41
G63	20.07	R127	20.10
E39	16.78	Y145	20.06
I41	12.92	D140	17.91
D65	7.77	L149	16.25
L64	5.40	L118	13.89
P105	5.28	G143	11.93
Y139	4.94	D132	10.41
A71	2.62	Y124	6.18
		P126	5.28

Table S5: Hess's cosine content analysis of three principal components (PC1-PC3) obtained from mass weighted covariance analysis.

ID	Cosine content
PC1	0.0586
PC2	0.0445
PC3	0.0722

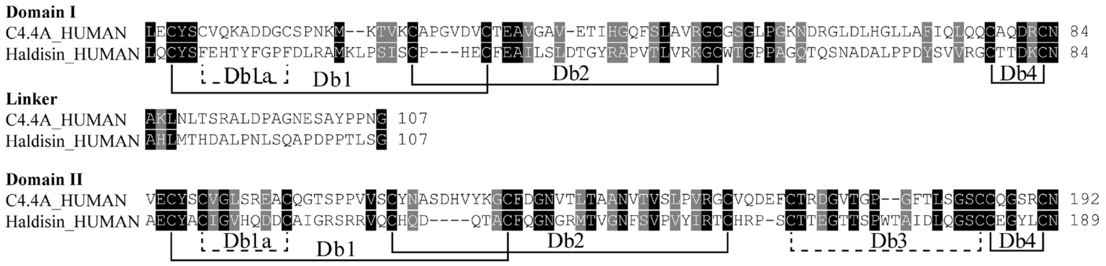

Figure S1. Sequence alignment of human C4.4A and human Haldisin. There is only a low degree of homology between C4.4A and Haldisin. Strictly conserved amino acids are show in black background. The alignment was done with the Clustal Omega [1] and then drew by BoxShade of ExPASy.

References

[1] Sievers F, Wilm A, Dineen D, Gibson TJ, Karplus K, Li W, et al. Fast, scalable generation of high-quality protein multiple sequence alignments using Clustal Omega. Molecular Systems Biology. 2011;7:539.

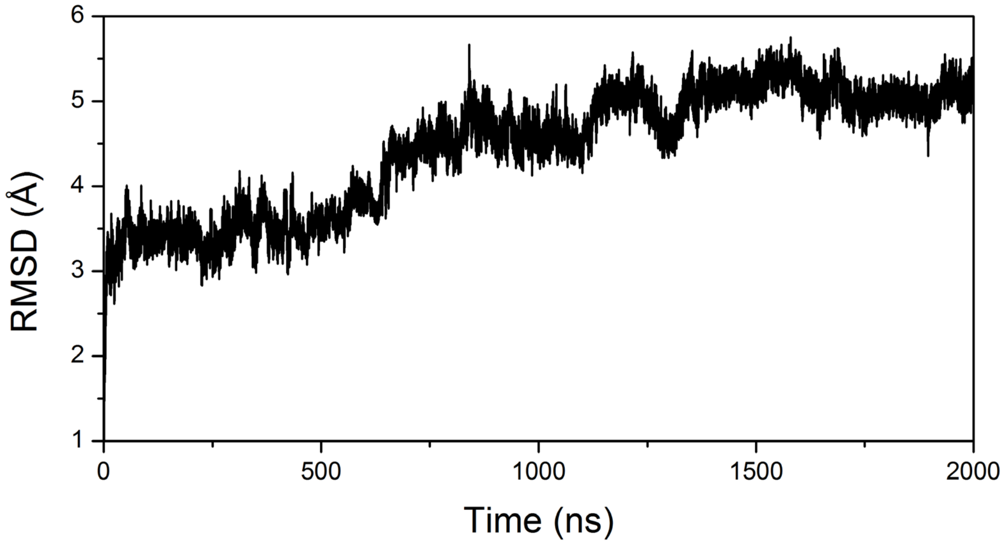

Figure S2. Backbone RMSD relative to the initial structure of Haldisin as a function of MD simulation time.
